# Imaging DivIVA dynamics using photo-convertible and activatable fluorophores in *Bacillus subtilis*

**DOI:** 10.3389/fmicb.2014.00059

**Published:** 2014-02-18

**Authors:** Juri N. Bach, Nadine Albrecht, Marc Bramkamp

**Affiliations:** Department of Biology I, Ludwig-Maximilians-UniversityMunich, Planegg-Martinsried, Germany

**Keywords:** division-site selection, DivIVA, dendra2, PA-GFP, photoconversion, photoactivation

## Abstract

Most rod-shape model organisms such as *Escherichia coli* or *Bacillus subtilis* utilize two inhibitory systems for correct positioning of the cell division apparatus. While the nucleoid occlusion system acts in vicinity of the nucleoid, the Min system was thought to protect the cell poles from futile division leading to DNA-free miniature cells. The Min system is composed of an inhibitory protein, MinC, which acts at the level of the FtsZ ring formation. MinC is recruited to the membrane by MinD, a member of the MinD/ParA family of Walker-ATPases. Topological positioning of the MinCD complex depends on MinE in *E. coli* and MinJ/DivIVA in *B. subtilis*. While MinE drives an oscillation of MinCD in the *E. coli* cell with a time-dependent minimal concentration at midcell, the *B. subtilis* system was thought to be stably tethered to the cell poles by MinJ/DivIVA. Recent developments revealed that the Min system in *B. subtilis* mainly acts at the site of division, where it seems to prevent reinitiation of the division machinery. Thus, MinCD describe a dynamic behavior in *B. subtilis*. This is somewhat inconsistent with a stable localization of DivIVA at the cell poles. High resolution imaging of ongoing divisions show that DivIVA also enriches at the site of division. Here we analyze whether polar localized DivIVA is partially mobile and can contribute to septal DivIVA and vice versa. For this purpose we use fusions with green to red photoconvertible fluorophores, Dendra2 and photoactivatable PA-GFP. These techniques have proven very powerful to discriminate protein relocalization *in vivo*. Our results show that *B. subtilis* DivIVA is indeed dynamic and moves from the poles to the new septum.

## Introduction

Placement of protein complexes in bacterial cells can be accomplished by energy driven systems using ATP hydrolyzing enzymes of the MinD/ParA protein family (Lutkenhaus, 2012). The Min system has been identified in rod-shaped bacteria due to its characteristic mutant phenotype. Absence of Min components leads to aberrant division resulting in small, DNA-free cells, called miniature cells (Adler et al., 1967). Electron micrographs of *E. coli min* mutants revealed that minicells seem to occur close to cell poles and, hence, the Min system was thought to be an active mechanism to protect the cell poles from aberrant cytokinesis (Rothfield et al., 2005; Lutkenhaus, 2007). This hypothesis was built based on data derived from *E. coli* as model organism. In *E. coli* the Min system consists of three proteins MinC, MinD, and MinE (all encoded in the so-called *minB* locus) (De Boer et al., 1988; Akerlund et al., 1992). MinC is an actual inhibitor of FtsZ ring formation (Bi and Lutkenhaus, 1990; Marston and Errington, 1999), with FtsZ being the first division protein localized at the new division site (Bi and Lutkenhaus, 1991). MinD is a Walker-type ATPase that binds reversibly to the plasma membrane in its ATP-bound form (De Boer et al., 1991) and recruits MinC. The ATP hydrolysis cycle leading to reversible membrane-binding of MinD (and hence the MinCD complex) is stimulated by MinE (Zhao et al., 1995; Hu and Lutkenhaus, 2001). This simple feedback loop creates a robust oscillation of MinCDE in *E. coli* (Hu and Lutkenhaus, 1999; Raskin and De Boer, 1999a,b) that can also be reconstructed *in vitro* on supported membrane surfaces (Loose et al., 2008).

Phyogenetic analyses made clear that Min proteins are conserved in many bacterial species. Among many other species Min proteins have also been identified in *B. subtilis* (Reeve et al., 1973; Varley and Stewart, 1992; Lee and Price, 1993). *B. subtilis* lacks the topological determinant MinE, but a functional homolog was found in DivIVA (Cha and Stewart, 1997; Edwards and Errington, 1997; Marston et al., 1998). Initial localization studies with DivIVA suggested that DivIVA is localized to the cell poles and at sites of ongoing septation (Marston et al., 1998). DivIVA was considered to be stably attached to the pole regions (Edwards and Errington, 1997; Edwards et al., 2000) and an intrinsic affinity for negatively curved membrane has been shown (Lenarcic et al., 2009; Ramamurthi and Losick, 2009). DivIVA recruits MinD via a bridging protein, MinJ (Bramkamp et al., 2008; Patrick and Kearns, 2008). In contrast to *E. coli* where the Min oscillation would leave a time-based MinC minimum around midcell, allowing FtsZ to form a functional ring here, the *B. subtilis* DivIVA/MinCDJ system was considered to be more static (Errington et al., 2003; Adams and Errington, 2009). However, recently new data on Min protein localization in *B. subtilis* suggest that components of the Min system in *B. subtilis* may not be static at the cell poles, but relocate from cells poles to active septa (Gregory et al., 2008; Bramkamp and Van Baarle, 2009; Van Baarle and Bramkamp, 2010). Thus, the actual site of action for the Min system in vegetative *B. subtilis* is the septum, rather than the cell pole and likely reinitiation of cell division next to a recently completed septum is prevented by action of the Min proteins (Bramkamp et al., 2008; Gregory et al., 2008; Bramkamp and Van Baarle, 2009; Van Baarle and Bramkamp, 2010). While dynamic relocation of MinC has been clearly shown, potential dynamics of other Min components in *B. subtilis* remained unclear. A recent study on MinJ and DivIVA using super resolution light microscopy has suggested that DivIVA may indeed be static and not dynamic. However, it was shown in this study that DivIVA accumulations at the cell poles are reduced and simultaneously the DivIVA amount at the active division site increases (Eswaramoorthy et al., 2011). Using GFP fusion proteins it is difficult to distinguish between newly synthesized and dynamic proteins. We therefore used here photoactivatable PA-GFP and photoconvertible Dendra2 fusion constructs and monitored DivIVA distribution in actively growing *B. subtilis* cells. Our data indicate that *B. subtilis* DivIVA is indeed dynamic and molecules from the cell pole (old division site) contribute to the formation of new DivIVA assemblies at the site of ongoing division. Thus, the *B. subtilis* Min system is dynamically relocalized within the cell cycle. Our work also highlights the potential use of photoconvertible tools in bacterial cell biology.

## Materials and methods

### Strain construction

The strains, plasmids and oligonucleotides used in this study are listed in Tables 1–3, respectively. *E. coli* DH5α was used to amplify and maintain plasmids. DNA was digested by restriction enzymes (New England Biolabs) and all plasmids were verified by DNA sequencing. PA-GFP was amplified from pPAGFP-N1 using the primer pair PAGFP-pJPR1-f and PAGFP-pJPR1-r and Dendra2 was amplified with the primer pair Dendra-pJPR1-f and Dendra-pJPR1-r with pDendra2-N as template DNA. Both PCR products were cloned into pJPR1 resulting in pJPR1-Dendra and pJPR1-PAGFP. pJPR1-DivIVA-PAGFP and pJPR1-DivIVA-Dendra were constructed by amplifying *divIVA* from genomic DNA using the primer DivIVA-PAGFP-f and DivIVA-PAGFP-r or DivIVA-Dendra-f and DivIVA-Dendra-r. The PCR products were cloned into pJPR1-PAGFP resulting in pJPR1-DivIVA-PAGFP or into pJPR1-Dendra resulting in pJPR1-DivIVA-Dendra.

**Table 1:** **Oligonucleotides used in this study**.

**Oligonucleotide**	**Sequence**	**Restriction site**
DivIVA-Dendra-f	CATAAGCTTGGAGGTGGCATCATGCCATTAACG	HindIII
DivIVA-Dendra-r	ATGGGATCCTTCCTTTTCCTCAAATACAGC	BamHI
Dendra-pJPR1-f	CCGGATCCATGAACACCCCGGGAATTAACCTGATC	BamHI
Dendra-pJPR1-r	CCCACTAGTTTACCACACCTGGCTGGGCA	SpeI
DivIVA-PAGFP-f	CGTTAAGCTTTTTTTTCTCCATCTGTG	HindIII
DivIVA-PAGFP-r	GCGACTAGTTTCCTTTTCCTCAAA	SpeI
PAGFP-pJPR1-f	GCGACTAGTGTGAGCAAGGGCGAGGAGCT	SpeI
PAGFP-pJPR1-r	AATGCGGCCGCTTACTTGTACAGCTCGTC	NotI

**Table 2:** **Plasmids used in this study**.

**Plasmid**	**Characteristics**	**Reference/source**
pDendra2-N	*pUC ori, SV40 ori, PCMVIE, Kan^r^*	Evrogen
pPAGFP-N1	*pBR322 ori, f1 ori, PCMVIE, Kan^r^*	Addgene
pJPR1	*bla amyE3' cat Pxyl amyE5'*	Bramkamp et al., 2008
pJPR1-Dendra	*bla amyE3' cat Pxyl dendra amyE5'*	This work
pJPR1-PAGFP	*bla amyE3' cat Pxyl pagfp amyE5'*	This work
pJPR1-DivIVA-PAGFP	*bla amyE3' cat Pxyl divIVA_pagfp amyE5'*	This work
pJPR1-DivIVA-Dendra	*bla amyE3' cat Pxyl divIVA_dendra amyE5'*	This work

**Table 3:** ***B. subtilis* and *E. coli* strains used in this study**.

	**Strains**	**Genotype**	**Reference/source**
***B. subtilis***			
	168	*trpC2*	Laboratory collection
	BB008	*trpC2 amyE::(cam Pxyl-divIVA-pagfp)*	pJPR1-DivIVA-PAGFP –> 168
	BB009	*trpC2 amyE::(cam Pxyl-divIVA-dendra)*	pJPR1-DivIVA-Dendra –> 168
	1803	*divIVA::(P_divIVA_-gfp divIVA ^+^ cat)*	Thomaides et al., 2001
***E.coli***			
	DH5a	F^−^ Φ 80lacZM15 (*lacZYA-argF*)U169 *recA*1 *endA*1 *hsdR*17(rk^−^,mk^+^) *phoA supE*44 *thi*−1 *gyrA*96 *relA*1 λ ^−^	Invitrogen

### Growth conditions

*B. subtilis* was grown with aeration in flasks using suitable media (LB or CH). Antibiotics were used when appropriate (tetracycline 10 μg ml^−1^; chloramphenicol 5 μg ml^−1^kanamycin 5 μg ml^−1^). *B. subtilis* strains were always inoculated from fresh overnight cultures.

For time lapse microscopy of *B. subtilis* expressing DivIVA-GFP cells were grown in LB to an OD_600_ of 1. Cells were diluted 1:100 in fresh pre-warmed LB and mounted on pre-warmed 1% agarose pads supplemented with LB. To inhibit protein biosynthesis LB agarose pads were supplemented with kanamycin to a final concentration of 5 μg ml^−1^. The agar pads were sealed using paraffin (Sigma-Aldrich; 327212) and incubated 10 min at 37°C before microscopic analysis.

Cells expressing DivIVA-Dendra or DivIVA-PA-GFP were treated as described above with the exception that growth medium and agar pads were supplemented with 1% fructose and with varying concentrations between 0.1 and 1% xylose.

### Fluorescence microscopy

For time lapse microscopy images were taken on a Zeiss Axio Observer Z1 microscope equipped with a Hamamatsu OrcaR^2^ camera. A Plan-Apochromat 100×/1.4 Oil Ph3 objective (Zeiss) was used and GFP fluorescence was visualized with filterset 38 HE eGFP shift free (Zeiss). The microscope was equipped with an environmental chamber set to 37°C. Images were taken every 2 min. Digital images were acquired with Zen software (Zeiss).

For photo-activation, photo-conversion and FRAP experiments a Delta Vision Elite (GE Healthcare, Applied Precision) equipped with an Insight SSI™ illumination, an X4 laser module and a CoolSnap HQ2 CCD camera was used. Images were taken with a 100× oil PSF U-Plan S-Apo 1.4 NA objective or with a 60× oil PlanApo, NA. 1.42 objective.

A four color standard set InsightSSI unit with following excitation wavelengths (DAPI 390/18 nm FITC 475/28 nm, TRITC 542/27 nm, Cy5 632/22 nm); single band pass emission wavelengths (DAPI 435/48 nm, FITC 525/48 nm, TRITC 597/45 nm, Cy5 679/34 nm) and a suitable polychroic for DAPI/FITC/TRITC/Cy5 were used. GFP and Dendra2 (green version) were visualized using FITC settings and converted Dendra2 was imaged using the TRITC filter. Bleaching was performed using a 405 nm laser (50 mW) with 10% power and 0.01–0.05 s illumination. For photo-activation of PA-GFP a 405 nm laser (50 mW) with 30% power and 0.5 s illumination was used. For PA-GFP fluorescence detection a FITC filter set (see above) was used (100% power, 1 s). Photo-conversion of Dendra2 was performed using a 405 nm laser (50 mW) with 15% power and 0.05 s illumination was used. Green fluorescence of Dendra was monitored using a FITC specific filter set, a TRITC specific filter set (0.25 s illumination; 50% power) was used to detect the photo-converted, red -shifted Dendra.

Analysis of the images was performed using ImageJ 1.45 s. The corrected total fluorescence (CTF) was calculated according to following formula: CTF = Integrated Density—(Area of selected cell X Mean fluorescence of unspecific background readings) (Gavet and Pines, 2010). For FRAP experiments unspecific background was subtracted for every ROI (see above). The CTF of the septa was divided by the CTF of the whole cell. The respective quotient of the unbleached spot was always set as 1.

Final image preparation was done in Adobe Photoshop CS2 (Adobe Systems Incorporated). All imaging experiments were performed several times with biological replicates.

## Results

### Time lapse analysis of DivIVA-GFP

**Figure 1:**
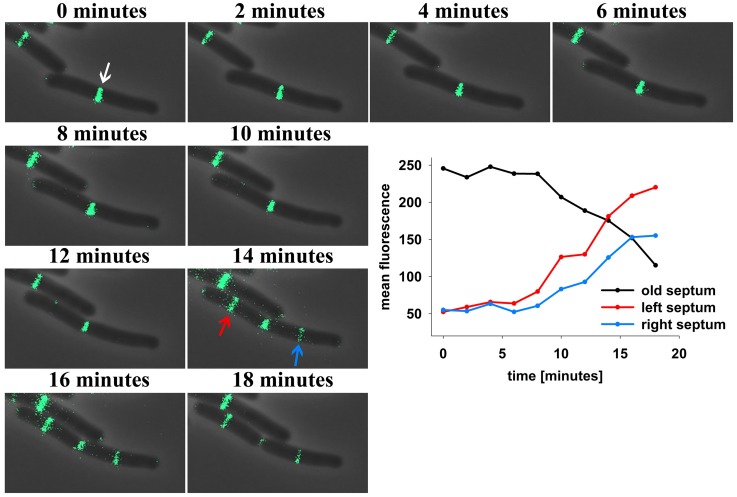
**Time lapse analysis reveals DivIVA-GFP dynamics in *B. subtilis***. Cells expressing DivIVA-GFP under its native promoter were grown on agarose slides supplemented with LB at 37°C and analyzed microscopically. Pictures were taken every 2 min. After division DivIVA-GFP is mostly located at midcell or at one pole (white arrow). After 14 min DivIVA-GFP is recruited to new forming septa (red and blue arrow) and fluorescence intensity at the old septa decreases stepwise. For plotting the fluorescence measured at the old and new formed septa ROIs of identical size were drawn and the mean fluorescence of every spot was calculated individually.

Subcellular localization of DivIVA in *B. subtilis* has been reported in various publications (Edwards and Errington, 1997; Thomaides et al., 2001; Hamoen and Errington, 2003; Perry and Edwards, 2004; Lenarcic et al., 2009; Oliva et al., 2010; Eswaramoorthy et al., 2011). Non-dividing cells show DivIVA accumulations at both cell poles, while actively dividing cells were reported to have DivIVA at the poles and at the site of ongoing septation. A recent study using 3D structured illumination revealed that DivIVA localizes in a ring-like fashion at the septum, while patches of DivIVA remain at the cell poles (Eswaramoorthy et al., 2011). The polar patches described by Eswaramoorthy and colleagues have apparently less DivIVA compared to the DivIVA concentration at the septum, and hence, the question remains whether DivIVA molecules may move from the cell pole to midcell. We reinvestigated DivIVA localization in growing cells of strain 1803 (Thomaides et al., 2001) using time lapse microscopy (Figure 1). DivIVA-GFP localization in exponentially growing cells seems to concentrate almost entirely at the septum. When the fluorescence intensity of DivIVA at midcell is quantified starting with fully matured septa over time a steady decrease indicates the loss of DivIVA molecules from the septum with ongoing division or pole formation (Figure 1). At the same time, new septa occurring on either side of the old septum gain DivIVA molecules as indicated by the strong increase in fluorescence. A simple and plausible explanation of this dynamic protein behavior would be the migration of polar DivIVA material to the new sites of division. It should be noted that the total fluorescence at the new septa exceeds the initial fluorescence at the cell poles (Figure 1). Using time lapse microscopy it is not possible to discriminate between proteolysis/new synthesis of DivIVA and dynamic relocalization of DivIVA.

### DivIVA localization is dynamic in *B. subtilis*

**Figure 2:**
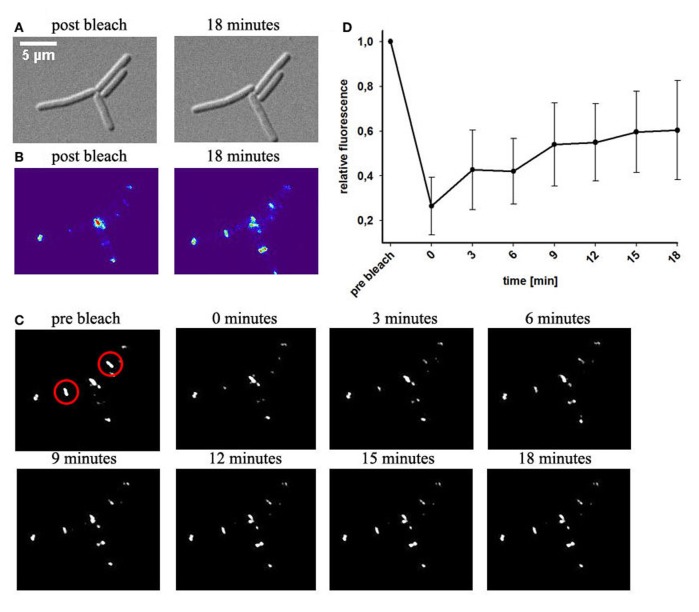
**Fluorescence recovery after photobleaching of DivIVA-GFP**. Cells expressing DivIVA-GFP under control of the native promoter were grown on agarose slides supplemented with LB. **(A)** DIC images of *B. subtilis* cells expressing DivIVA-GFP directly after a bleaching event and after 18 min are shown **(B)** Heat maps of the GFP signal and time lapse images indicate fluorescence distribution **(C)**. DivIVA-GFP was bleached (red circles) as described in material and methods. Pictures were taken every 3 min after the bleaching event. Images show GFP fluorescence. **(D)** Quantification of the recovery rate of bleached spots; *n* = 6.

Protein mobility *in vivo* can be quantified with fluorescence recovery after photo-bleaching (FRAP). We bleached part of the DivIVA-GFP signal at one cell pole or at the septum and measured reoccurrence of GFP signals. Within few minutes the majority of the signal recovered in the bleached area (Figures 2A–D). This recovery is in contrast to results published earlier (Eswaramoorthy et al., 2011) where FRAP experiments were reported to have only little recovery. However, images in that publication reveal already slight recovery after 1 min (Eswaramoorthy et al., 2011). In order to rule out the possibility that recovery of fluorescence might exclusively be due to new synthesis of DivIVA-GFP, we blocked protein biosynthesis (see material and methods) before performing FRAP experiments. Strikingly, recovery of DivIVA-GFP in cells with inhibited protein biosynthesis was almost identical to the FRAP experiments without inhibitor (Figure S1). These results support that DivIVA dynamically reassembles at new division sites with material recruited from cell poles.

**Figure 3:**
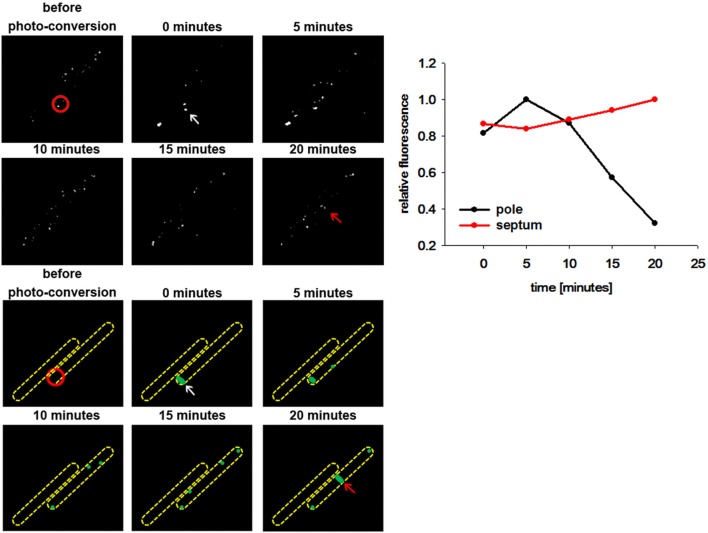
**DivIVA-PA-GFP is dynamically recruited from the cell pole to the septa**. DivIVA-PA-GFP fluorescence was imaged before photo-activation using DIC and FITC specific filters. Photoactivation was performed using a laser at 405 nm (red circle). After photoactivation DivIVA-PA-GFP (white arrow) is localized at the cell pole. Although, the signal gets more diffuse over time, accumulation at a new septum after 20 min becomes evident (red arrow). A cartoon of DivIVA-PA-GFP dynamics is drawn below. The relative fluorescence of the pole (white arrow) and the new formed septa (red arrow) was measured. The relative fluorescence of the according spots were calculated (CTF was calculated and the highest CTF of all spots were set as 1) and plotted. For every time point spots were chosen individually.

In order to unambiguously determine whether DivIVA molecules at new division site recruits material from old divisions (cell poles) we constructed a *B. subtilis* strain encoding for a DivIVA-PA-GFP construct. The photoactivatable GFP is non-fluorescent when the proteins are synthesized. Upon activation with a laser flash, PA-GFP is activated and the classical GFP fluorescence is readily observed (Patterson and Lippincott-Schwartz, 2002). Photoactivation of DivIVA-PA-GFP was achieved with a 0.5 s laser pulse of 405 nm (see material and methods). We converted PA-GFP at the pole region of growing cells and immediately after photoactivation green fluorescence was observed (Figure 3). Over time the green fluorescence was redistributed in the cell and accumulated at midcell position, while a clear decrease in fluorescence at the cell poles was observed. This behavior mimicked the DivIVA-GFP dynamics seen in the FRAP experiments. Control experiments indicated that imaging PA-GFP after photoactivation using the InsightSSI light source did not yield any conversion (data not shown), ruling out that new fluorescent material was generated during the time lapse imaging process. These data clearly suggest that DivIVA molecules are dynamic and material from old division sites (cell poles) contribute to the DivIVA assemblies at active division sites. Efficient activation of PA-GFP in bacteria needed a significant input of energy. Although we confirmed cell viability by cell growth, we realized that cells with efficient PA-GFP activation often died or grew slower, likely due to phototoxic effects.

**Figure 4:**
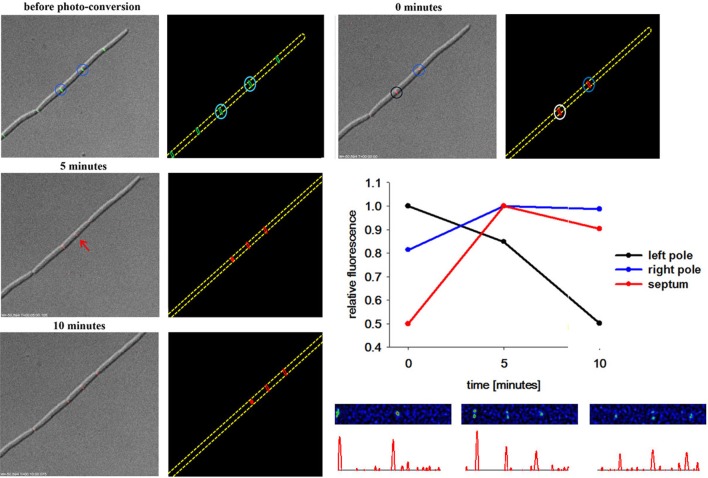
**DivIVA-Dendra2 is dynamically recruited from the cell pole to the septa**. DivIVA-Dendra2 fluorescence (green and red) was imaged before photoconversion using DIC, FITC, or TRITC specific filters. After photoconversion using a 405 nm laser (cyan circles) only red fluorescence (TRITC) and DIC was monitored to prevent additional photoconversion. After 5 min DivIVA-Dendra2 is recruited from the place of photoconversion (black and blue circle) to new septa forming (red arrow). A cartoon of the photoconversion is shown. The relative fluorescence of the left pole (black circle), the right pole (blue circle), and the new formed septa (red arrow) was measured. The relative fluorescence of the corresponding spots were calculated (CTF was calculated and the highest CTF of each spot was set as 1) and plotted. For every time point spots were chosen individually. Heat maps and corresponding histograms are shown below.

Milder conditions are apparently needed to avoid phototoxic effects. Therefore, we turned our attention to a photoconvertible fluorophore, Dendra2 (Gurskaya et al., 2006; Chudakov et al., 2007). Dendra2 is a monomeric protein with a green-to-red photoconversion upon blue light exposure (Gurskaya et al., 2006). Dendra2 was reported to fold efficiently in bacteria and its photostability makes it ideal for long-term protein tracking (Gurskaya et al., 2006). We have constructed a DivIVA-Dendra2 and analyzed localization of the translational fusion protein in growing cells (Figure 4). Green fluorescence was readily observed at cell poles and septa, undistinguishable from the DivIVA-GFP fusion. DivIVA-Dendra was converted by a very fast (0.05 s) 405 nm laser flash. Immediately after the conversion time lapse analysis revealed the generation of a red fluorophore at the site of conversion. Since imaging of green fluorescence at 488 nm exposure slowly, but significantly convert more DivIVA-Dendra2 from green to red, we only followed the red signal. Clearly, the red, converted DivIVA redistributed and accumulated over time at a new septum that was formed (Figure 4 and Figure S2). Control experiments using the same imaging conditions without laser event revealed no photoconversion (data not shown). Thus, DivIVA-Dendra2 which was converted at an ongoing site of division or a cell pole redistributes to new division sites.

## Discussion

**Figure 5:**
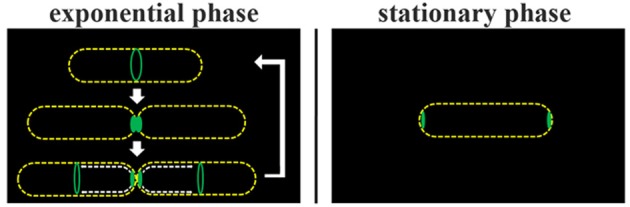
**Model of DivIVA dynamics in *B. subtilis***. During cytokinesis DivIVA rings at constricting septa collapse into foci/patches (Eswaramoorthy et al., 2011). Exponentially growing *B. subtilis* cells redeploy at least a fraction of DivIVA molecules from old division sites (cell poles) to nascent septa (broken white lines). Only in non-dividing cells DivIVA is clearly seen as accumulation at both cell poles.

Regulation of division site selection is a primary problem in cell biology. Although intensively studied the exact mechanisms of septum placement is not even fully understood in simple rod-shaped bacteria. Particularly puzzling was the finding that the Gram negative bacterium *E. coli* used an oscillating Min system to position its cytokinetic machinery while a geometrically similar rod, *B. subtilis* seemed to have a static Min system tethered to cell poles by DivIVA. Recent observations have shed some doubt on the static nature of the *B. subtilis* Min localization (Bramkamp et al., 2008; Gregory et al., 2008; Van Baarle and Bramkamp, 2010). However, it remained unclear whether only the MinCD complex might be dynamic and whether the topological factors MinJ and DivIVA are static components. An attempt to use super-resolution microscopy showed that DivIVA accumulates at the sites of ongoing divisions and reduces to a patch-like focus at the old cell pole (Eswaramoorthy et al., 2011). However, FRAP experiments in this study led to the suggestion that DivIVA molecules are not dynamically re-localized to new sites of division. We revisited this question based on time lapse analysis with quantitative analysis of DivIVA-GFP in growing *B. subtilis*. Quantification of GFP signals revealed that the loss of GFP at the old division site and accumulation of GFP at the new sites of division runs in parallel (Figure 1). In accord with this, FRAP experiments in presence and absence of antibiotics inhibiting protein biosynthesis revealed a slow recovery of DivIVA. A fundamental difference to the fast oscillation of the topological factor in the *E. coli* Min system, MinE, which oscillates within seconds, DivIVA dynamics are within minutes (Figures 1, 2). This slow redistribution of DivIVA could also be explained by a localized proteolysis at the old pole and assembly of newly synthesized material at the new sites of septation. The data presented here cannot exclude localized proteolysis of DivIVA at the cell poles. In order to demonstrate clearly a dynamic component of the spatio-temporal DivIVA distribution with the cell we used photoactivatable and photoconvertible fluorophores in this study. PA-GFP and Dendra2 are stably activated or converted by a laser pulse and the protein portion that was activated/converted can be tracked precisely over time. With this technique we could clearly show that DivIVA molecules from previous division sites contribute to the formation of new septa (Figures 3, 4). Hence, our data indicate that DivIVA in *B. subtilis* is not static but dynamic, leaving old division sites (cell poles) and accumulating at nascent septa (Figure 5). Cells in stationary phase or otherwise non-growing cells, have DivIVA at both cell poles, however, actively growing cells, reduce the DivIVA concentration at the old septa/poles fast and redeploy DivIVA molecules to build DivIVA rings at active division sites (Figure 5). Our data contribute to the new ideas of Min functioning in cell division in *B. subtilis*. In contrast to a long standing hypothesis the Min system in *B. subtilis* is not statically protecting the old poles from aberrant division, but rather prevents reinitiation of septation close to active septa (Bramkamp et al., 2008; Gregory et al., 2008; Van Baarle and Bramkamp, 2010; Eswaramoorthy et al., 2011). The avoidance of reinitiation seems to be regulated at different levels. The MinCD system likely prevents reassembly of mature FtsZ rings, while proteolysis of free FtsL may prevent reassembly of membrane components of the divisome (Wadenpohl and Bramkamp, 2010).

Interestingly, a dynamic DivIVA/Min system in *B. subtilis* provokes a couple of questions about the molecular mechanism, by which this dynamic behavior is regulated. DivIVA was shown to bind to curved membrane areas (Lenarcic et al., 2009; Ramamurthi and Losick, 2009). A molecular bridging model for the formation of DivIVA assemblies at curved membranes has been presented (Lenarcic et al., 2009). The molecular bridging model suggested that DivIVA complexes with fewer contacts might diffuse away from the membrane. In dividing *B. subtilis* cells this could simply be triggered by pole formation where the cell pole rounds up, after daughter cell separation. However, other cellular factors might play a role in DivIVA dynamics as well. Several groups have reported phosphorylation of DivIVA homologs in actinobacteria and firmicutes (Kang et al., 2005, 2008; Beilharz et al., 2012; Hempel et al., 2012; Saalbach et al., 2013). Maybe reversible phosphorylation of DivIVA could be a cue to shuttle the protein between a membrane attached and soluble from. The function and dynamics of the *B. subtilis* Min system apparently needs revisiting and will likely bring up some surprises.

### Conflict of interest statement

The authors declare that the research was conducted in the absence of any commercial or financial relationships that could be construed as a potential conflict of interest.

## References

[B1] Adams D. W. Errington J. (2009). Bacterial cell division: assembly, maintenance and disassembly of the Z ring. Nat. Rev. Microbiol. 7, 642–653 10.1038/nrmicro2198 19680248

[B2] Adler H. I. Fisher W. D. Cohen A. Hardigree A. A. (1967). MINIATURE *Escherichia coli* cells deficient in DNA. Proc. Natl. Acad. Sci. U.S.A. 57, 321–326 10.1073/pnas.57.2.321 16591472 PMC335508

[B3] Akerlund T. Bernander R. Nordstrom K. (1992). Cell division in *Escherichia coli minB* mutants. Mol. Microbiol. 6, 2073–2083 10.1111/j.1365-2958.1992.tb01380.x 1406249

[B4] Beilharz K. Novakova L. Fadda D. Branny P. Massidda O. Veening J. W. (2012). Control of cell division in *Streptococcus pneumoniae* by the conserved Ser/Thr protein kinase StkP. Proc. Natl. Acad. Sci. U.S.A. 109, E905–E913 10.1073/pnas.1119172109 22431591 PMC3326482

[B5] Bi E. Lutkenhaus J. (1990). Interaction between the *min* locus and *ftsZ*. J. Bacteriol. 172, 5610–5616 2211499 10.1128/jb.172.10.5610-5616.1990 PMC526872

[B6] Bi E. F. Lutkenhaus J. (1991). FtsZ ring structure associated with division in *Escherichia coli*. Nature 354, 161–164 10.1038/354161a0 1944597

[B7] Bramkamp M. Emmins R. Weston L. Donovan C. Daniel R. A. Errington J. (2008). A novel component of the division-site selection system of *Bacillus subtilis* and a new mode of action for the division inhibitor MinCD. Mol. Microbiol. 70, 1556–1569 10.1111/j.1365-2958.2008.06501.x 19019154

[B8] Bramkamp M. Van Baarle S. (2009). Division site selection in rod-shaped bacteria. Curr. Opin. Microbiol. 12, 683–688 10.1016/j.mib.2009.10.002 19884039

[B9] Cha J. H. Stewart G. C. (1997). The *divIVA* minicell locus of *Bacillus subtilis*. J. Bacteriol. 179, 1671–1683 9045828 10.1128/jb.179.5.1671-1683.1997 PMC178881

[B10] Chudakov D. M. Lukyanov S. Lukyanov K. A. (2007). Using photoactivatable fluorescent protein Dendra2 to track protein movement. Biotechniques 42, 553–563 10.2144/000112470 17515192

[B11] De Boer P. A. Crossley R. E. Hand A. R. Rothfield L. I. (1991). The MinD protein is a membrane ATPase required for the correct placement of the *Escherichia coli* division site. EMBO J. 10, 4371–4380 1836760 10.1002/j.1460-2075.1991.tb05015.x PMC453190

[B12] De Boer P. A. Crossley R. E. Rothfield L. I. (1988). Isolation and properties of *minB*, a complex genetic locus involved in correct placement of the division site in *Escherichia coli*. J. Bacteriol. 170, 2106–2112 2834323 10.1128/jb.170.5.2106-2112.1988 PMC211093

[B13] Edwards D. H. Errington J. (1997). The *Bacillus subtilis* DivIVA protein targets to the division septum and controls the site specificity of cell division. Mol. Microbiol. 24, 905–915 10.1046/j.1365-2958.1997.3811764.x 9219999

[B14] Edwards D. H. Thomaides H. B. Errington J. (2000). Promiscuous targeting of *Bacillus subtilis* cell division protein DivIVA to division sites in *Escherichia coli* and fission yeast. EMBO J. 19, 2719–2727 10.1093/emboj/19.11.2719 10835369 PMC212753

[B15] Errington J. Daniel R. A. Scheffers D. J. (2003). Cytokinesis in bacteria. Microbiol. Mol. Biol. Rev. 67, 52–65 10.1128/MMBR.67.1.52-65.2003 12626683 PMC150516

[B16] Eswaramoorthy P. Erb M. L. Gregory J. A. Silverman J. Pogliano K. Pogliano J. (2011). Cellular architecture mediates DivIVA ultrastructure and regulates min activity in *Bacillus subtilis*. MBio 2:e00257 10.1128/mBio.00257-11 22108385 PMC3225972

[B47] Gavet O. Pines J. (2010). Progressive activation of CyclinB1-Cdk1 coordinates entry to mitosis. Dev. Cell 18, 533–543 10.1016/j.devcel.2010.02.013 20412769 PMC3325599

[B17] Gregory J. A. Becker E. C. Pogliano K. (2008). *Bacillus subtilis* MinC destabilizes FtsZ-rings at new cell poles and contributes to the timing of cell division. Genes Dev. 22, 3475–3488 10.1101/gad.1732408 19141479 PMC2607072

[B18] Gurskaya N. G. Verkhusha V. V. Shcheglov A. S. Staroverov D. B. Chepurnykh T. V. Fradkov A. F. (2006). Engineering of a monomeric green-to-red photoactivatable fluorescent protein induced by blue light. Nat. Biotechnol. 24, 461–465 10.1038/nbt1191 16550175

[B19] Hamoen L. W. Errington J. (2003). Polar targeting of DivIVA in *Bacillus subtilis* is not directly dependent on FtsZ or PBP 2B. J. Bacteriol. 185, 693–697 10.1128/JB.185.2.693-697.2003 12511520 PMC145330

[B20] Hempel A. M. Cantlay S. Molle V. Wang S. B. Naldrett M. J. Parker J. L. (2012). The Ser/Thr protein kinase AfsK regulates polar growth and hyphal branching in the filamentous bacteria Streptomyces. Proc. Natl. Acad. Sci. U.S.A. 109, E2371–E2379 10.1073/pnas.1207409109 22869733 PMC3435184

[B21] Hu Z. Lutkenhaus J. (1999). Topological regulation of cell division in *Escherichia coli* involves rapid pole to pole oscillation of the division inhibitor MinC under the control of MinD and MinE. Mol. Microbiol. 34, 82–90 10.1046/j.1365-2958.1999.01575.x 10540287

[B22] Hu Z. Lutkenhaus J. (2001). Topological regulation of cell division in *E*. coli. spatiotemporal oscillation of MinD requires stimulation of its ATPase by MinE and phospholipid. Mol. Cell 7, 1337–1343 10.1016/S1097-2765(01)00273-8 11430835

[B23] Kang C. M. Abbott D. W. Park S. T. Dascher C. C. Cantley L. C. Husson R. N. (2005). The *Mycobacterium tuberculosis* serine/threonine kinases PknA and PknB: substrate identification and regulation of cell shape. Genes Dev. 19, 1692–1704 10.1101/gad.1311105 15985609 PMC1176007

[B24] Kang C. M. Nyayapathy S. Lee J. Y. Suh J. W. Husson R. N. (2008). Wag31, a homologue of the cell division protein DivIVA, regulates growth, morphology and polar cell wall synthesis in mycobacteria. Microbiology 154, 725–735 10.1099/mic.0.2007/014076-0 18310019

[B25] Lee S. Price C. W. (1993). The *minCD* locus of *Bacillus subtilis* lacks the *minE* determinant that provides topological specificity to cell division. Mol. Microbiol. 7, 601–610 10.1111/j.1365-2958.1993.tb01151.x 8459776

[B26] Lenarcic R. Halbedel S. Visser L. Shaw M. Wu L. J. Errington J. (2009). Localisation of DivIVA by targeting to negatively curved membranes. EMBO J. 28, 2272–2282 10.1038/emboj.2009.129 19478798 PMC2690451

[B27] Loose M. Fischer-Friedrich E. Ries J. Kruse K. Schwille P. (2008). Spatial regulators for bacterial cell division self-organize into surface waves *in vitro*. Science 320, 789–792 10.1126/science.1154413 18467587

[B28] Lutkenhaus J. (2007). Assembly dynamics of the bacterial MinCDE system and spatial regulation of the Z ring. Annu. Rev. Biochem. 76, 539–562 10.1146/annurev.biochem.75.103004.142652 17328675

[B29] Lutkenhaus J. (2012). The ParA/MinD family puts things in their place. Trends Microbiol. 20, 411–418 10.1016/j.tim.2012.05.002 22672910 PMC3436946

[B30] Marston A. L. Errington J. (1999). Selection of the midcell division site in *Bacillus subtilis* through MinD-dependent polar localization and activation of MinC. Mol. Microbiol. 33, 84–96 10.1046/j.1365-2958.1999.01450.x 10411726

[B31] Marston A. L. Thomaides H. B. Edwards D. H. Sharpe M. E. Errington J. (1998). Polar localization of the MinD protein of *Bacillus subtilis* and its role in selection of the mid-cell division site. Genes Dev. 12, 3419–3430 10.1101/gad.12.21.3419 9808628 PMC317235

[B32] Oliva M. A. Halbedel S. Freund S. M. Dutow P. Leonard T. A. Veprintsev D. B. (2010). Features critical for membrane binding revealed by DivIVA crystal structure. EMBO J. 29, 1988–2001 10.1038/emboj.2010.99 20502438 PMC2892376

[B33] Patrick J. E. Kearns D. B. (2008). MinJ (YvjD) is a topological determinant of cell division in *Bacillus subtilis*. Mol. Microbiol. 70, 1166–1179 10.1111/j.1365-2958.2008.06469.x 18976281

[B34] Patterson G. H. Lippincott-Schwartz J. (2002). A photoactivatable GFP for selective photolabeling of proteins and cells. Science 297, 1873–1877 10.1126/science.1074952 12228718

[B35] Perry S. E. Edwards D. H. (2004). Identification of a polar targeting determinant for *Bacillus subtilis* DivIVA. Mol. Microbiol. 54, 1237–1249 10.1111/j.1365-2958.2004.04363.x 15554965

[B36] Ramamurthi K. S. Losick R. (2009). Negative membrane curvature as a cue for subcellular localization of a bacterial protein. Proc. Natl. Acad. Sci. U.S.A. 106, 13541–13545 10.1073/pnas.0906851106 19666580 PMC2726380

[B37] Raskin D. M. De Boer P. A. (1999a). MinDE-dependent pole-to-pole oscillation of division inhibitor MinC in *Escherichia coli*. J. Bacteriol. 181, 6419–6424 10515933 10.1128/jb.181.20.6419-6424.1999 PMC103778

[B38] Raskin D. M. De Boer P. A. (1999b). Rapid pole-to-pole oscillation of a protein required for directing division to the middle of Escherichia coli. Proc. Natl. Acad. Sci. U.S.A. 96, 4971–4976 10.1073/pnas.96.9.4971 10220403 PMC21801

[B39] Reeve J. N. Mendelson N. H. Coyne S. I. Hallock L. L. Cole R. M. (1973). Minicells of *Bacillus subtilis*. J. Bacteriol. 114, 860–873 4196259 10.1128/jb.114.2.860-873.1973 PMC251848

[B40] Rothfield L. Taghbalout A. Shih Y. L. (2005). Spatial control of bacterial division-site placement. Nat. Rev. Microbiol. 3, 959–968 10.1038/nrmicro1290 16322744

[B41] Saalbach G. Hempel A. M. Vigouroux M. Flärdh K. Buttner M. J. Naldrett M. J. (2013). Determination of phosphorylation sites in the DivIVA cytoskeletal protein of *Streptomyces coelicolor* by targeted LC-MS/MS. J. Proteome Res. 12, 4187–4192 10.1021/pr400524d 23905541 PMC3787806

[B42] Thomaides H. B. Freeman M. El Karoui M. Errington J. (2001). Division site selection protein DivIVA of *Bacillus subtilis* has a second distinct function in chromosome segregation during sporulation. Genes Dev. 15, 1662–1673 10.1101/gad.197501 11445541 PMC312724

[B43] Van Baarle S. Bramkamp M. (2010). The MinCDJ system in *Bacillus subtilis* prevents minicell formation by promoting divisome disassembly. PLoS ONE 5:e9850 10.1371/journal.pone.0009850 20352045 PMC2844427

[B44] Varley A. W. Stewart G. C. (1992). The *divIVB* region of the *Bacillus subtilis* chromosome encodes homologs of *Escherichia coli* septum placement (*minCD*) and cell shape (*mreBCD*) determinants. J. Bacteriol. 174, 6729–6742 1400225 10.1128/jb.174.21.6729-6742.1992 PMC207348

[B45] Wadenpohl I. Bramkamp M. (2010). DivIC stabilizes FtsL against RasP cleavage. J. Bacteriol. 192, 5260–5263 10.1128/JB.00287-10 20644139 PMC2944526

[B46] Zhao C. R. De Boer P. A. Rothfield L. I. (1995). Proper placement of the *Escherichia coli* division site requires two functions that are associated with different domains of the MinE protein. Proc. Natl. Acad. Sci. U.S.A. 92, 4313–4317 10.1073/pnas.92.10.4313 7753804 PMC41934

