# Supplementary material for: Imaging DivIVA dynamics using photo-convertible and activatable fluorophores in Bacillus subtilis
Source: Front Microbiol. 2014 Feb 18;5:59. doi: 10.3389/fmicb.2014.00059 (PMC3927310; doi:10.3389/fmicb.2014.00059)
Supplement: Supplementary file 1 [file DataSheet1.PDF]

Supplemental Material

Imaging DivIVA dynamics using photo-convertible and activatable fluorophores in *Bacillus subtilis*

Juri Niño Bach, Nadine Albrecht and Marc Bramkamp[‡]

Department of Biology I, Ludwig-Maximilians-University, Munich, Großhaderner Str. 2-4, 82152 Planegg-Martinsried, Germany.

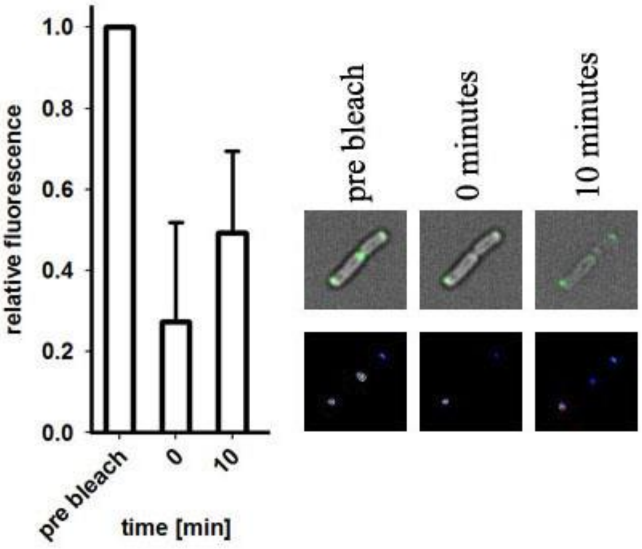

Fig. S1: FRAP of DivIVA-GFP with inhibited protein biosynthesis. Cells expressing DivIVA-GFP under control of the native promoter were placed on agarose slides supplemented with LB and kanamycin (5 µg ml^{-1}). The recovery of the bleached spot was calculated as described in material and methods and plotted. Images show a merge of bright field and GFP fluorescence (upper row) and a heat map of the GFP signal (lower row). Number of analyzed cells = 5.

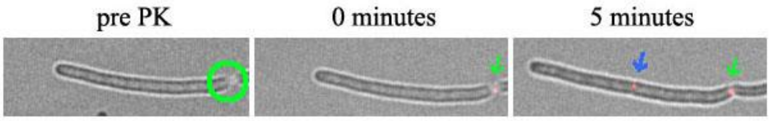

Figure S2: **DivIVA-Dendra2 dynamically relocalizes from poles to septa.** DivIVA-Dendra2 fluorescence (red) was imaged before photo-conversion using DIC and TRITC specific filters. After photo-conversion using a 405nm laser (green circle) red fluorescence (TRITC) and bright field was monitored. After 5 minutes DivIVA-Dendra2 is recruited from the place of photo-conversion (green arrow) to new septa forming (blue arrow).
